# Supplementary material for: Effect of the Size and Shape of Ho, Tm:KLu(WO4)2 Nanoparticles on Their Self-Assessed Photothermal Properties
Source: Nanomaterials (Basel). 2021 Feb 14;11(2):485. doi: 10.3390/nano11020485 (PMC7918361; doi:10.3390/nano11020485)
Supplement: Supplementary file 1 [file nanomaterials-11-00485-s001.pdf]

SUPPORTING INFORMATION

Effect of the size and shape of Ho, Tm:KLu(WO_4)$_2$ nanoparticles in their self-assessed photothermal properties

Albenc Nexha, Maria Cinta Pujol,* Joan J. Carvajal, Francesc Díaz and Magdalena Aguiló

Universitat Rovira i Virgili, Departament Química Física i Inorgànica, Física i Cristal·lografia de Materials i Nanomaterials (FiCMA-FiCNA)-EMaS, Campus Sescelades, E-43007, Tarragona, Spain

*mariacinta.pujol@urv.cat

Table S1. Summary of all the experimental parameters applied in the microwave assisted and conventional heating solvothermal method for the synthesis of KLu(WO_4)$_2$ nanocrystals. Under the column "Label", the text "MW" or "CA" refers to the type of reaction applied, "MW" stands for microwave assisted and "CA" for conventional autoclave heating solvothermal method. OLAC:OLAM presents the molar ratio between the organic surfactants. T_R, t_R, T_C and t_C represent the reaction temperature, reaction time, calcination temperature and calcination time of the reaction methodology applied, respectively.

Label	Precursors	OLAC:OLAM	K:Lu:W	T_R (K)	t_R (h)	T_C(K)	t_C (h)	Product
MW-1	$Lu(NO_3)_3 \cdot H_2O$, K_2WO_4	1:1	4:1:2	453	3	1023	2	KLuW
MW-2	$Lu(NO_3)_3 \cdot H_2O$,K_2WO_4	1:1	4:1:2	423	3	1023	2	KLuW
MW-3	$Lu(NO_3)_3 \cdot H_2O$,K_2WO_4	1:1	4:1:2	393	3	1023	2	KLuW
MW-4	$Lu(NO_3)_3 \cdot H_2O$,K_2WO_4	1:1	4:1:2	453	2	1023	2	KLuW
MW-5	$Lu(NO_3)_3 \cdot H_2O$,K_2WO_4	1:1	4:1:2	453	1	1023	2	KLuW
MW-6	$Lu(NO_3)_3 \cdot H_2O$,K_2WO_4	1:1	4:1:2	373	0.5	1023	2	KLuW
MW-7	$Lu(NO_3)_3 \cdot H_2O$,K_2WO_4	1:1	4:1:2	343	0.5	1023	2	KLuW
MW-8	$Lu(NO_3)_3 \cdot H_2O$,K_2WO_4	1:1	4:1:2	303	0.5	1023	2	KLuW
MW-9	$Lu(NO_3)_3 \cdot H_2O$,K_2WO_4	1:4	4:1:2	453	3	1023	2	KLuW
MW-10	$Lu(NO_3)_3 \cdot H_2O$,K_2WO_4	-	4:1:2	453	3	1023	2	K_2WO_4 + Lu_2O_3
MW-11	$Lu(Ac)_3 \cdot H_2O$,K_2WO_4	1:1	4:1:2	453	3	1023	2	KLuW
MW-12	$Lu(NO_3)_3 \cdot H_2O$,K_2WO_4	1:1	1:1:1/2	453	3	1023	2	Lu_2O_3
MW-13	$Lu(NO_3)_3 \cdot H_2O$,K_2WO_4	1:1	1:1:2	453	3	1023	2	Unknown
MW-14	$Lu(NO_3)_3 \cdot H_2O$,K_2WO_4	1:1	4:1:2	453	3	973	2	KLuW
MW-15	$Lu(NO_3)_3 \cdot H_2O$,K_2WO_4	1:1	4:1:2	453	3	923	2	KLuW + Others
MW-16	$Lu(NO_3)_3 \cdot H_2O$,K_2WO_4	1:1	4:1:2	453	3	873	2	KLuW + Others
MW-17	$Lu(NO_3)_3 \cdot H_2O$,K_2WO_4	1:1	4:1:2	453	3	1023	1.5	KLuW
MW-18	$Lu(NO_3)_3 \cdot H_2O$,K_2WO_4	1:1	4:1:2	453	3	1023	1	KLuW + Others
MW-19	$Lu(NO_3)_3 \cdot H_2O$,K_2WO_4	1:1	4:1:2	453	3	1023	0.5	KLuW + Others
CA-1	$Lu(NO_3)_3 \cdot H_2O$,K_2WO_4	1:1	4:1:2	453	3	1023	2	KLuW
CA-2	$Lu(NO_3)_3 \cdot H_2O$,K_2WO_4	1:1	4:1:2	423	3	1023	2	KLuW
CA-3	$Lu(NO_3)_3 \cdot H_2O$,K_2WO_4	1:1	4:1:2	393	3	1023	2	KLuW
CA-4	$Lu(NO_3)_3 \cdot H_2O$,K_2WO_4	1:1	4:1:2	453	12	1023	2	K_2WO_4 + Lu_2O_3
CA-5	$Lu(NO_3)_3 \cdot H_2O$,K_2WO_4	1:1	4:1:2	453	6	1023	2	KLuW
CA-6	$Lu(NO_3)_3 \cdot H_2O$,K_2WO_4	1:1	4:1:2	453	2	1023	2	KLuW
CA-7	$Lu(NO_3)_3 \cdot H_2O$,K_2WO_4	1:1	4:1:2	453	1	1023	2	KLuW
CA-8	$Lu(NO_3)_3 \cdot H_2O$,K_2WO_4	1:1	4:1:2	453	0.5	1023	2	KLuW
CA-9	$Lu(NO_3)_3 \cdot H_2O$,K_2WO_4	1:1	4:1:2	453	0.25	1023	2	KLuW
CA-10	$Lu(NO_3)_3 \cdot H_2O$,K_2WO_4	1:1	4:1:2	453	0.16	1023	2	KLuW
CA-11	$Lu(NO_3)_3 \cdot H_2O$,K_2WO_4	1:1	4:1:2	453	0.08	1023	2	KLuW

Table S2. Average size of the most relevant final products obtained from the MW and CA solvothermal synthesis, calculated from TEM images and Debye-Scherrer equation.

Label	Size from TEM images (nm)	Size from Debye-Scherrer equation (nm)
MW-1	12	19
MW-2	82	53
MW-3	164	68
MW-4	33	37
MW-5	111	67
MW-9	275	77
MW-11	41	48
CA-1	16	28
CA-2	132	64
CA-3	145	67
CA-5	1167	80
CA-6	142	60
CA-7	171	60
CA-8	212	68
CA-9	215	68
CA-10	221	71
CA-11	416	80

Section S1. Microwave-assisted solvothermal synthesis of $KLu(WO_4)_2$ nanocrystals

1.1. The effect of the reaction temperature on the microwave-assisted solvothermal synthesis of $KLu(WO_4)_2$ nanocrystals.

The influence of the reaction temperature in the microwave-assisted (hereafter MW) solvothermal synthesis of the monoclinic KLuW was analyzed in terms of crystalline phase and morphology of the obtained seeds and the final products. Keeping constant the molar ratio of OLAC:OLAM=1:1, the precursors $Lu(NO_3)_3{\cdot}H_2O$ and K_2WO_4, and the reaction time, first, we tested the effect of the reaction temperature, setting it in successive experiments at 453 K, 423 K and 393 K, labeled as MW-1, MW-2 and MW-3. Table S1 summarizes all the experimental parameters used for each synthesis. Figure S1 (a) and (b) shows the XRPD pattern of the seeds and the final products obtained as described.

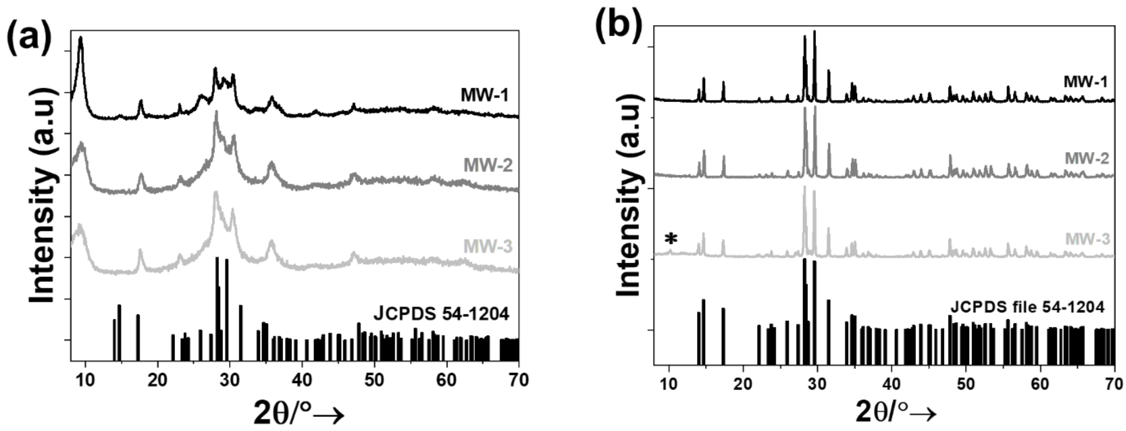

Figure S1. XRPD patterns of the products obtained by the MW solvothermal method for the synthesis of KLuW nanocrystal performed at different reaction temperatures for 3 h: (a) seeds and (b) final products obtained by the calcination of the seeds at 1023 K during 2 h (Refer to Table 1 for full experimental details). The reference pattern of KLuW (JCPDS 54-1204) is included for comparison. The diffraction peaks that do not belong to the KLuW phase in the final products are marked with *.

As shown in Figure S1 (a), the seeds are crystalline, but they are not the monoclinic crystalline KLuW, because the diffraction pattern does not match. The X-ray patterns of the seeds exhibit just a few significant broaden peaks in all of the three reaction temperatures which difficult its identification. We believe that they might be formed by a mixture of different compounds, among which KLuW can also be present with a low degree of crystallinity, as some of the more prominent peaks observed in the patterns almost coincide with the position of the most intense peaks for this compound in the reference pattern included also in this figure (JCPDS file 54-1204).
The XRPD patterns of the final products obtained at all reaction temperatures (Figure S1 (b)) are attributed to the monoclinic crystalline phase of KLuW (JCPDS file 54-1204), space group *C*2/*c*. The temperature of the reaction has not a significant influence on the crystalline structure of the final products obtained. However, the X-ray diffraction of the product obtained at the lowest temperature analyzed, shows one additional extra diffraction peak with a very low intensity, indicated in the Figure S1 (b) with asterisk. Since only one peak was observed with a very low intensity, it was impossible to identify to which compound it would belong, testing reaction precursors and the composition of the seeds.
Apparently, the seeds obtained at 453 K show a more homogeneous and defined morphology as shown in the histogram of the length of the nanorods. The nanorods have a wide length distribution ranging from 100 nm up to 800 nm, with a higher concentration of nanorods with a length around 320 nm and a diameter around 36 nm. By decreasing the reaction temperature to 423 K, it resulted in an increase of the average diameter of the seeds, in the range of 100 nm (see Figure S2 (b)). Further decrease of the reaction temperature to 393 K, produced an aggregation of the particles and the appearance of some particles with a new morphological habit consisting of spheres (see Figure S2 c). Figure S2 (d)-(f) shows the TEM images obtained of the final products. As it can be observed, the final product (see Figure S2 d) originated from the calcination of the seeds with a more defined morphology obtained at 453 K (see Figure S2 (a)), show a narrower size distribution and smaller particles than the final products derived from the seeds obtained at other reaction temperatures. The size of the nanocrystals increased drastically with the decrease of the temperature of the reaction. Thus, the final product obtained at 453 K, had an average size distribution of 12 nm (see Table S2), the product obtained at 423 K had an average size of 82 nm and the product obtained at 393 K has the largest average size with a value of 164 nm (see Table S2). In addition, the calculation of the average crystallite sizes with the Debye-Scherrer equation, confirms the size of monoclinic KLuW nanocrystals (see Table S2). The crystalline habit for the final products shown in Figure S2, is irregular, with no defined crystalline faces and a tendency to be ellipsoids.

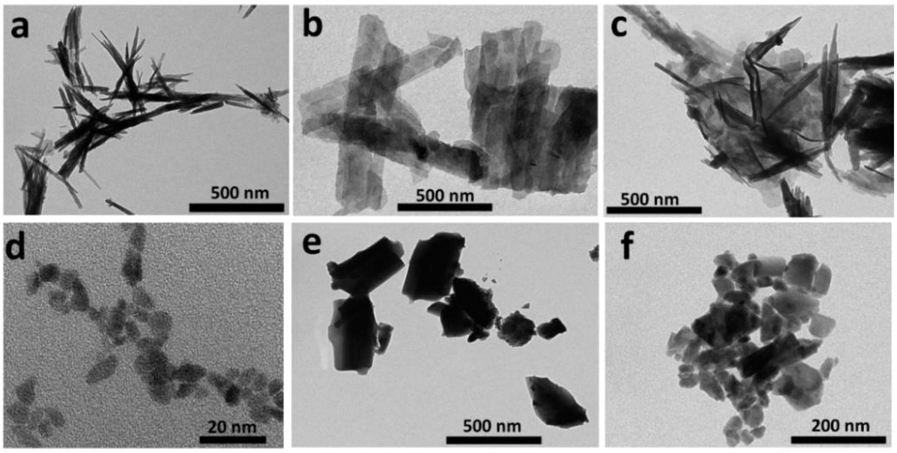

Figure S2. TEM images of the seeds and the final products, respectively obtained by the MW solvothermal method, synthesized at different reaction temperatures during 3 h: (a) and (d) MW-1, (b) and (e) MW-2, (c) and (f) MW-3. In all cases, the final product is obtained by the calcination of the seeds at 1023K during 2h.

1.2. Effect of the time of the reaction on the microwave-assisted solvothermal synthesis of $KLu(WO_4)_2$ nanocrystals

We also analyzed the effect of the time of the reaction on the MW solvothermal synthesis. For that, the MW reaction temperature was set to 453 K and the time of the reaction was tuned, keeping the other parameters unchanged

(experiments summarized in Table S1). The seeds and the final products of the reactions at 3 h (labeled as MW-1), 2 h (labeled as MW-4) and 1 h (labeled as MW-5) were analyzed via XRPD and TEM. The XRPD patterns obtained are shown in Figure S3 (a) and (b).

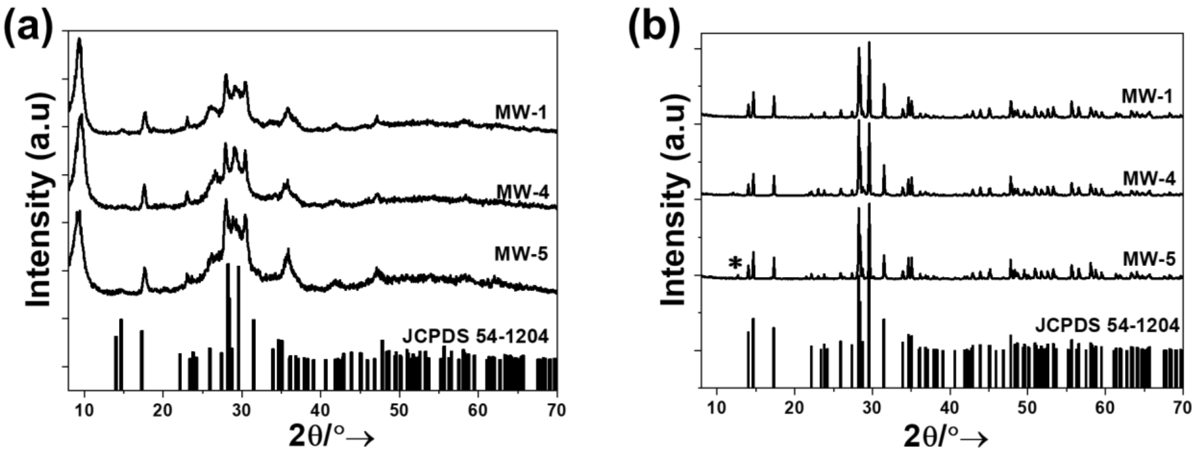

Figure S3. XRPD patterns of the products obtained by the MW solvothermal synthesis method performed at 453 K and at different times: (a) seeds and (b) final products. The reference pattern of KLuW (JCPDS 54-1204) is included for comparison.

As in the previous case, the seeds show a similar XRPD patterns, with no important effect of the reaction time on them. Again, it is difficult to define the compound that forms the seeds, although KLuW cannot be disregarded, as can be seen from the reference diffraction pattern. The final products obtained, are all formed basically by KLuW. However, as the reaction time decreases, some additional peaks with a low intensity appear, although it was impossible to determine at which crystalline phase they belong.

Figure S4 (a)-(f) shows the TEM images of the seeds and the final products obtained by the MW solvothermal method setting the reaction temperature at 453 K and changing the reaction time. Decreasing the reaction time from 3 h to 1 h, implies changes in the morphology of the seeds in the sequence of nanorods-rectangular plates-aggregated nanorods and nanoplates. As can be seen by the contrast in color in the images, the nanorods obtained at 3 h (see Figure S4 (a)) are thicker than the rectangular plates obtained after 2 h of reaction (see Figure S4 (b)). Finally, at 1 h, in the mixture obtained of nanorods and nanoplates (see Figure S4 (c)), it can be seen that the nanorods are thicker than the nanoplates. Taking into account the pure morphological habits of the reaction time of 3 h and 2 h (excluding here the morphological mixture of the 1 h reaction), the length and the width of the seeds increases with the decrease of the reaction time. The effect of the time of the reaction influences also the morphology and the size of the final products. They exhibit morphology close to a sphere and their sizes range from 12 nm up to 111 nm with the decrease of the reaction time from 3 h to 1 h (see Table S2).

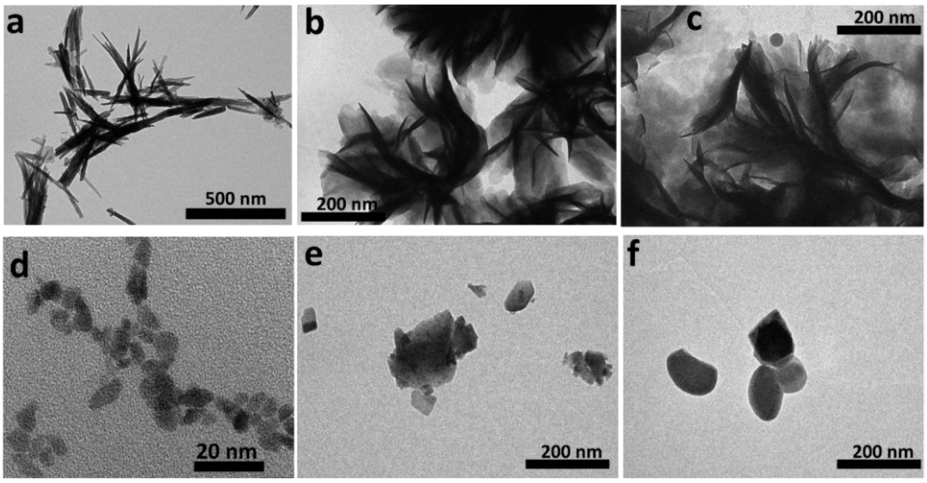

Figure S4. TEM images of the seeds and the final products for the KLuW synthesized via MW solvothermal method at 453 K and different reaction times: a) and d) MW-1, b) and e) MW-4, c) and f) MW-5.

Additionally, other synthesis were performed and analyzed structurally via XRPD (only the final products) using lower temperatures of the reaction, such as 373 K, 343 K and 303 K at 0.5 h as the reaction time, labeled as MW-6, MW-7 and MW-8, respectively. The patterns of the final products of the synthesis when the temperature of the reaction was 373 K, 343 K and 303 K are shown in Figure S5.

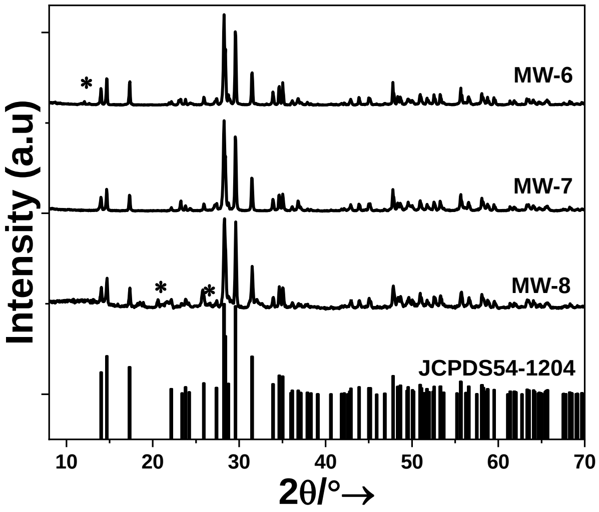

Figure S5. XRPD patterns of final products for the KLuW synthesized via MW solvothermal method using MW-6, MW-7 and MW-8 reaction conditions. The reference pattern of the m-KLuW (JCPDS 54-1204) is included for comparison. We have marked with *, peaks that do not belong to the KLuW monoclinic crystalline phase.

Although the major part of the peaks of pattern for all final products obtained at 373 K, 343 K and 303 K can be attributed to the KLuW, the final product obtained at 303 K show a large number of additional diffraction peaks indicated by an asterisk. As we have seen discussed up to now, using as reaction temperature 453 K and as reaction time 3 h, the final products correspond to KLuW crystalline phase, without additional peaks and with a narrower size distribution. Therefore, we established these two parameters as optimum for the synthesis of KLuW via MW solvothermal method.

1.3. The effect of the organic surfactants on the microwave-assisted solvothermal synthesis of $KLu(WO_4)_2$ nanocrystals

We wanted to analyze the effect of the ratio of organic surfactants on the morphology and the crystalline structure of the seeds and the final products. When an excess amount of OLAM (labeled as MW-9), respect to OLAC (4 times) was provided to the reaction mixture, the XRPD pattern of the seeds and of the final product does not exhibit significant changes in comparison with those obtained in a reaction with equimolar ratio of surfactants (Figure S6 (a)). Additional low intensity peaks are observed in the case of the XRPD pattern of final product (Figure S6 (b)).

However, the morphology of the seeds obtained by using an excess of OLAM in the reaction (shown in Figure S7 (a)), changed significantly comparing with the nanorods obtained from an equimolar ratio of OLAC:OLAM (see Figure S2 (a)). They exhibit a flower-like type morphology composed of curved nanorods with a smaller width of approximately 20 nm, compared to the seeds obtained in the equimolar ratio reaction. The final product obtained with an excess of OLAM (see Figure S7 (b)), does not exhibit a regular morphology and shows particles with sizes in the range of 275 nm (see Table S2). To verify the significant role of the surfactants in the synthesis reaction, we tested the reaction without the presence of these organic surfactants (labeled as MW-10).

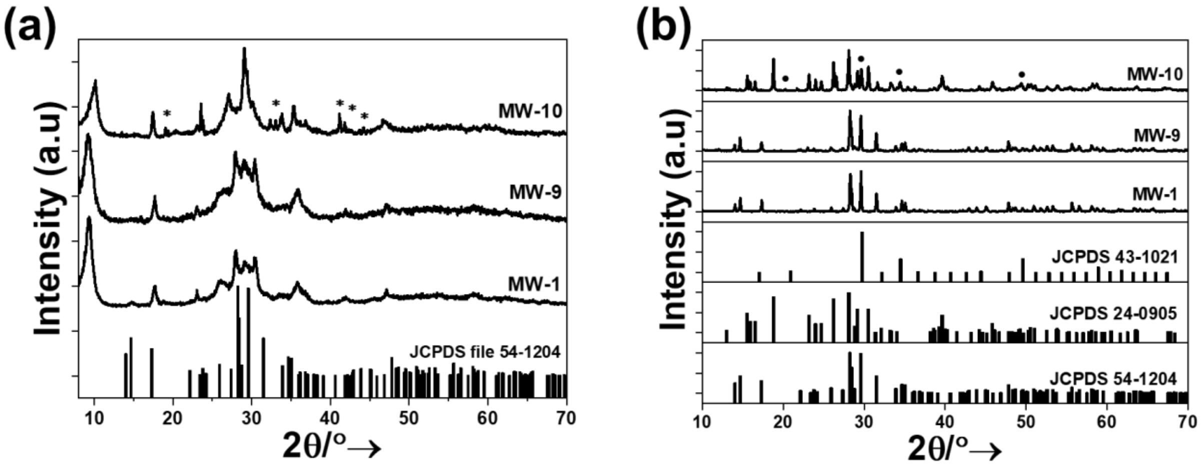

Figure S6. XRPD pattern of the products obtained via MW solvothermal method by varying the ratio of the organic surfactants: (a) seeds and (b) final products. The XRPD reference patterns of the KLuW (JCPDS file 54-1204), K_2WO_4 (JCPDS file 24-0905), and Lu_2O_3 (JCPDS file 43-1021) are included for comparison. Marked in asterisk, the new diffraction peaks that appeared on the seeds and final product when no organic surfactants were used in the reaction. Marked with black spheres, the diffraction peaks assigned to the cubic Lu_2O_3 phase for the final product.

Figure S6 (a) and (b) show the XRPD patterns of the seeds and the final products obtained when oleic acid and oleylamine were not introduced in the reaction solution, respectively, whereas the morphological features are presented in Figure S7 (c) and (d). As can be seen in Figure S6 (b), in the absence of the organic surfactants, a mixture between monoclinic K_2WO_4 (JCPDS file 24-0905) and cubic Lu_2O_3 (JCPDS file 43-1021) was obtained. An even more complex trend is observed in the XRPD patterns of the seeds (Figure 6a), apparently there is a mixture of compounds, taking into account all the diffraction peaks observed in all the seeds obtained until now. The TEM images revealed that both, the seeds (Figure S7 (c)) and the final products (Figure S7 (d)), obtained without the presence of organic surfactants in the reaction, exhibited an irregular morphology, with agglomerated nanoparticles and in the case of the seeds, very different from the nanorods or nanoplates observed in the previous experiments and discrete nanoparticles in the case of the final product.

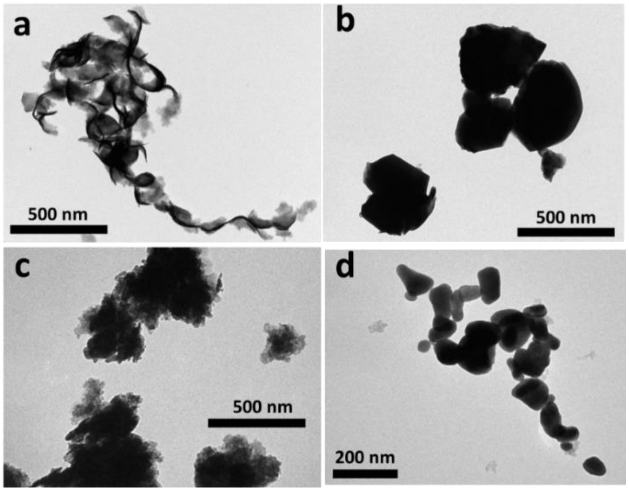

Figure S7. TEM images of seeds and final products synthesized via MW solvothermal method, modifying the concentration of the organic surfactants in the reaction solution: (a) and (b) MW-9, and (c) and (d) MW-10, used.

1.4. The effect of the precursors on the microwave-assisted solvothermal synthesis of $KLu(WO_4)_2$ nanocrystals

In this section, the aim is to test the effect of reagents used for the synthesis of KLuW and their concentration in the seeds and final products crystalline phases of the MW solvothermal reaction. Our synthetic approach is built of three types of precursors: the lutetium precursors, the potassium precursors and the tungsten precursors.

Initially, the lutetium precursor was changed from lutetium (III) nitrate hydrate to lutetium (III) acetate hydrate (labelled as MW-11), keeping the other parameters unchanged, including here the K:Lu:W ratio set at 4:1:2 (as in the case of lutetium (III) nitrate). The morphology of the seeds resembles the nanorods synthesized using lutetium (III) nitrate hydrate as precursor (see Figure S8 (a)), with similar lengths, except that they show an increase in their widths to approximately 100 nm. The TEM image of the final product reveal an irregular morphology with an average size distribution of around 41 nm (see Table S2). The XRPD pattern of the seeds is similar to that one obtained from the lutetium (III) nitrate reaction (see Figure S1 (a) and Figure S9 (a)) and the final product's crystalline phase was still attributed to the monoclinic KLuW (see Figure S9 (b)). Thus, the change of lutetium precursor has no effect on the formation of monoclinic KLuW phase. The next step was to check the influence of the K_2WO_4 precursor, thus, using 0.15 mmol K_2WO_4 and 0.3 mmol of Lu(III) precursor (either nitrate or acetate), we check what effect would have introducing into our reaction a lower amount of K_2WO_4, applying a ratio of K:Lu:W=1:1:1/2 (labelled as MW-12). The XRPD results for the seeds and final product are shown in Figure S9 (a) and (b). The results obtained for this K:Lu:W ratio reveal that the seeds are amorphous and the final product do not match with KLuW, but with the cubic Lu_2O_3 (JCPDS file 43-1021).

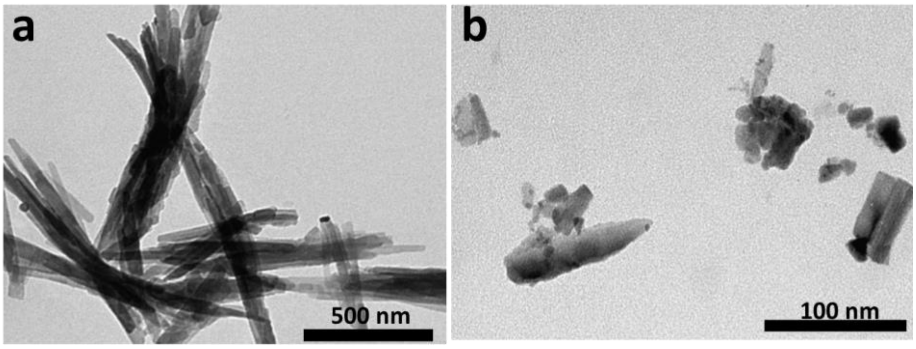

Figure S8. TEM images of the: (a) seeds and (b) final product synthesized via the MW solvothermal method using lutetium (III) acetate hydrate (reaction MW-11).

In a last experiment, following the stoichiometry of the desired final product $KLu(WO_4)_2$, we applied a ratio of K:Lu:W=1:1:2 (labeled as MW-13). To apply this stoichiometry, it is needed to use a different precursor for K and for W. Thus, we incorporated 0.3 mmol of Lu (III) precursor, 0.15 mmol of K_2CO_3 and 0.6 mmol of $(NH_4)_2WO_4$, having the desired ratio K:Lu:W=1:1:2. The introduction of K_2CO_3 and $(NH_4)_2WO_4$ is a typical combination used to synthesize monoclinic KLuW via the modified sol-gel Pechini method Clearly, the XRPD pattern of the seeds (see Figure S9 (a)) significantly changed from that of the K:Lu:W=4:1:2 ratio. The XRPD pattern of the final product does not match also with the KLuW crystalline phase and due to its complexity it was impossible to deduce to which compound/compounds it corresponds (see Figure S9 (b)).

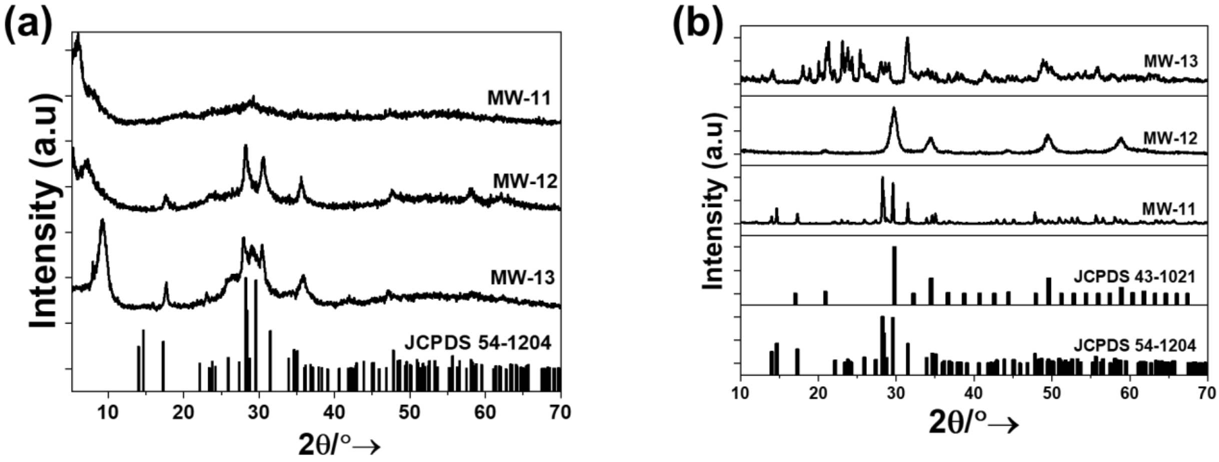

Figure S9. XRPD patterns of: (a) the seeds and (b) the final products obtained by changing or tuning the precursor reagents and their concentrations.

A possible explanation to why the initial reagent ratios K:Lu:W=1:1:2 and K:Lu:W=1:1:1/2 did not form the desired monoclinic KLuW phase, could be related to the fact that in order to allow the organic surfactants (OLAC, OLAM) to control the nucleation and growth of the nanocrystals, one should form a supersaturated solution, a critical condition for the formation of the first nucleus and as a consequence also of the final product. Since we are providing a lower amount of K^+ and W into our reaction mixture, this would imply no reactivity and block of the supersaturation conditions and formation of the desired crystalline phases for the seeds and final product. From this section, we conclude that the optimal precursor ratio for the synthesis of KLuW via the MW solvothermal method is K:Lu:W=4:1:2, using a single combined potassium and tungsten precursor (K_2WO_4).

1.5. Optimization of the calcination post treatment

We observed the effect of the calcination temperature on the crystalline phase formed in the final product of MW solvothermal reaction synthesis. To describe in more detail, the physical process that take place during the calcination step, transforming the seeds into the monoclinic KLuW crystalline phase, a careful Differential Thermal Analysis (DTA) and Thermal Gravimetric Analysis (TGA) of the seeds obtained by the MW solvothermal method was performed. The DTA and TGA analysis of the seeds and their interpretations, are presented in Figure 1 (b) of the manuscript.

The transformation from the unknown crystalline phase of the seeds to the monoclinic KLuW is covering the range of temperatures from around 973 K to 1173 K, so, to better define the appropriate calcination temperature, several experiments were performed applying different calcination temperatures during a constant time of 2 h. The calcination temperature was tuned from 1023 K (labeled as MW-1), 973 K (labeled as MW-14), 923 K (labeled as MW-15) and 873 K (labeled as MW-16). All experimental details assigned to the tuning of the calcination temperature are presented in Table S1. The XRPD patterns of the final products obtained are shown in Figure S10. At 1023 K, we obtained pure KLuW (see Figure S1 (b)), but when the calcination temperature is lower, the diffraction patterns resemble to that offered in Figure S1 (c), corresponding to the transition from the seeds to KLuW, and curiously the structure is almost identical to the temperature analyzed, proofing that these phases are formed below 973 K. According to these results, we established the optimal calcination temperature as 1023 K.

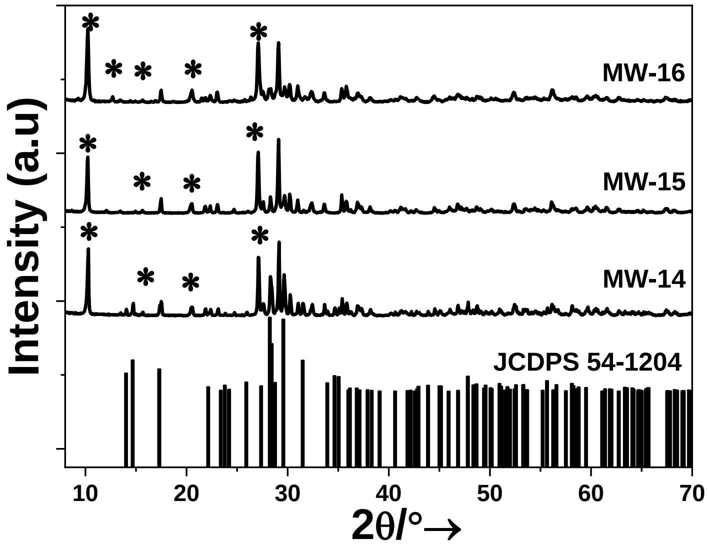

Figure S10. XRPD pattern of the products obtained via the MW solvothermal method on the synthesis of KLuW using different calcination temperatures.

Having established the calcination temperature, now we can tune the calcination time keeping the calcination temperature constant at 1023 K. The crystalline structure of the obtained products are shown in Figure S11. For

the calcination times at 2 h (MW-1) and 1.5 h (labeled as MW-17), the phase obtained belongs to monoclinic KLuW. However, for shorter calcination times (1 h (labeled as MW-18) and 0.5 h (labeled as MW-19)), a mixture between monoclinic KLuW and an unknown compound is observed. According to these results, we establish the optimal calcination time in between 1.5 h and 2 h for a calcination temperature of 1023 K.

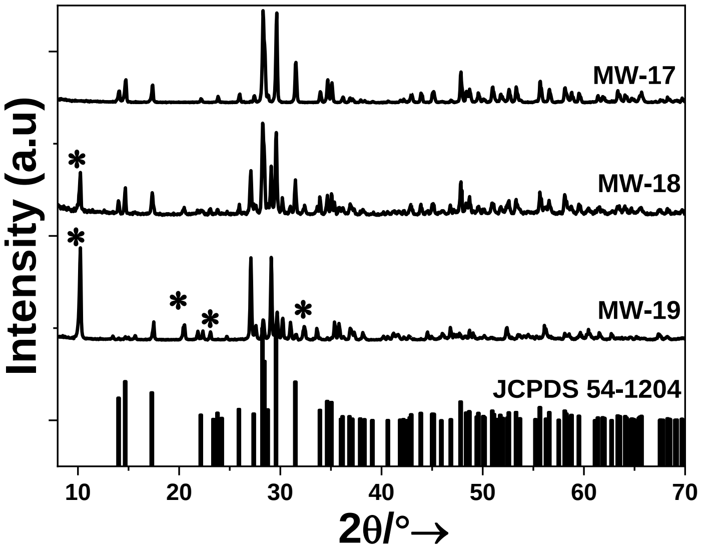

Figure S11. XRPD pattern of the products obtained by the MW solvothermal method for the synthesis of KLuW obtained at different calcination times when the calcination temperature was set at 1023 K. In black dots, marked the additional peaks not assigned to monoclinic KLuW.

All the experiments performed to optimize the synthesis of KLuW nanocrystals through the MW solvothermal method are summarized in Table S1. According to these results, the optima parameters for the synthesis of KLuW through this method are: use of $Lu(NO_3)_3{\cdot}H_2O$ and K_2WO_4 as precursors for Lu^{3+}, K^+ and WO_4^{2-}, use of an equimolar ratio of the organic surfactants OLAC and OLAM, perform the reaction at 453 K during 3 h and perform the calcination at 1023 K during 1.5 h to 2 h calcination temperature and time.

Section S2. Conventional autoclave solvothermal synthesis of $KLu(WO_4)_2$ nanocrystals

2.1. Effect of the conventional heating using metallic autoclave solvothermal synthesis of $KLu(WO_4)_2$ nanocrystals

We tested which is the effect of the reaction temperature in the conventional heating autoclave (CA) starting from the optimum temperature for the MW solvothermal method (453 K) (labeled as CA-1) and reducing it to 423 K (labeled as CA-2) and 393 K (labeled as CA-3). All the other parameters for the CA solvothermal method are summarized in Table S1. We tested if there is any effect of the use of the conventional heating autoclave (CA) in the solvothermal synthesis of the KLuW nanocrystals in comparison with the MW heating. The reaction temperatures analyzed were 453 K as the optimum one in the MW assisted method, and also lower temperatures were tested, as 423 K and 393 K. The other experimental parameters of the synthesis were the same as the optimized ones in the MW solvothermal approach. The obtained results were structurally and morphologically investigated, as in the previous sections.

The XRPD patterns of the seeds and the final products for the CA approach were similar to those obtained via the MW solvothermal method. The XRPD pattern of the seeds obtained through the CA solvothermal method at different reaction temperatures do no differ one from another, they exhibit the same XRPD pattern, the same broadening behavior, and we could not assign the XRPD pattern to any crystalline phase (see Figure S12 (a)). The XRPD pattern of the final products obtained at these different reaction temperatures could be identified as pure monoclinic KLuW in all cases, regardless of the reaction temperature applied (see Figure S12 (b)). However, some

additional XRPD diffraction peaks that do not belong to the monoclinic KLuW could be observed in the XRPD pattern of the product obtained at 393 K.

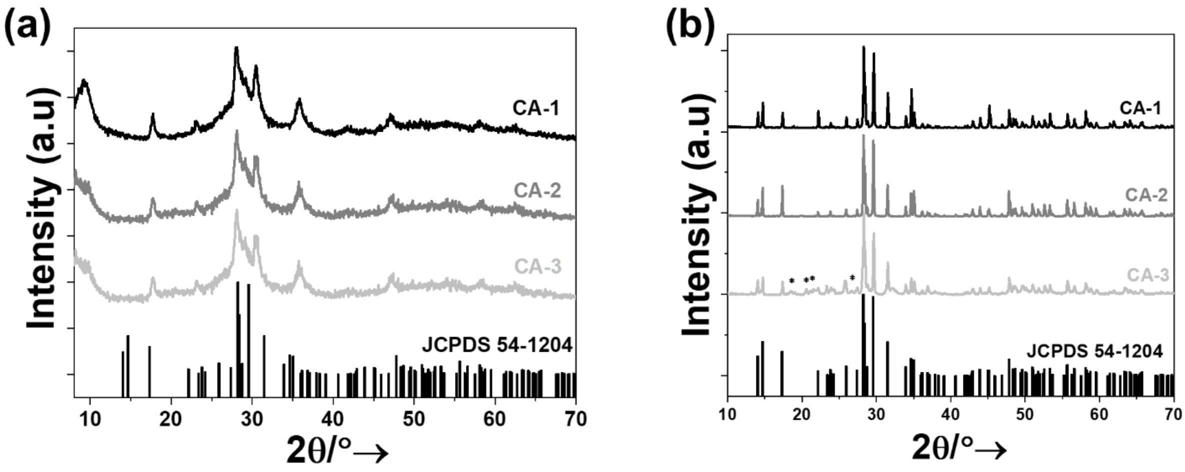

Figure S12. XRPD patterns obtained via the CA solvothermal method for the synthesis of KLuW performed at different reaction temperatures for 3 h: (a) seeds, and (b) final products. In all cases, the final product is obtained by the calcination of the seeds at 1023 K during 2 h. The reference pattern of KLuW (JCPDS 54-1204) is included for comparison. Marked with asterisk the diffraction peaks observed in the patterns that could not be attributed to monoclinic KLuW.

From the morphological point of view (see Figure S13), almost all the seeds obtained, exhibited an irregular shape and tendency to aggregation. The seeds of the reaction at 453 K, show a better defined and homogeneous morphology with an urchin-like shape with an average length of 585 nm. By decreasing the temperature down to 423 K and 393 K, it resulted in an increase of the tendency to aggregation with lengths approximately of 4.5 μm (see Figure S13 (b)) and the formation of structures with no defined shape (see Figure S13 (c)).

KLuW nanoparticles obtained via the CA reaction at 453 K show a small size and a more regular shape with an average size distribution of 16 nm (see Table S2). By decreasing the reaction temperature, it had the same effect on the final products, as it was discussed in the case of the seeds. Thus, when the reaction was performed at 423 K, it produced nanoparticles in the range of 132 nm, and when the reaction was performed at 393 K, the nanoparticles obtained had an average size of 326 nm (see Table S2).

According to these results, we established the 453 K as the optimal temperature for the preparation of the pure monoclinic KLuW by the CA solvothermal method, because we obtained smaller and more discrete nanoparticles then in the other temperatures.

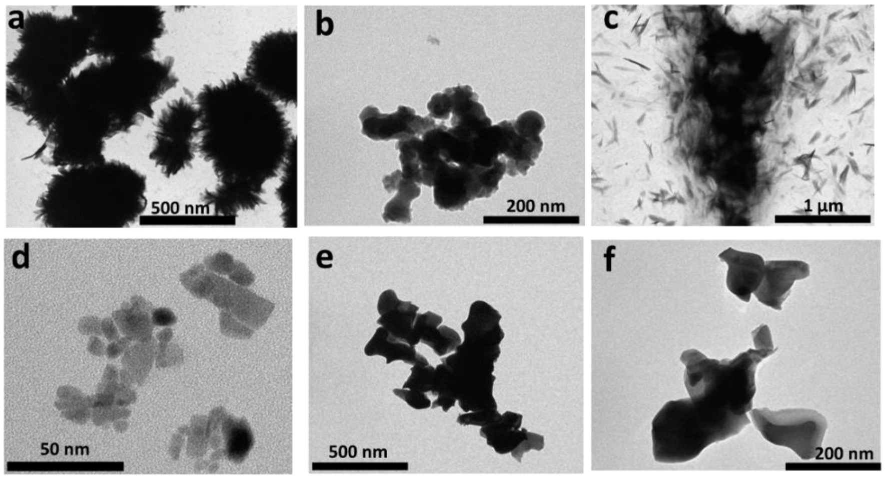

Figure S13. TEM images of the seeds and the final products obtained by the CA solvothermal method for the synthesis KLuW at different reaction temperatures during 3 h: (a) and (d) CA-1, b) and (e) CA-2, (c) and (f) CA-3, respectively. In

all cases, the final products are obtained by the calcination of the seeds at 1023 K during 2 h, (g) Lognormal length distribution of the final product presented in (d).

2.2. Effect of the reaction time on the CA solvothermal synthesis of $KLu(WO_4)_2$ nanocrystals.

Taking into account the results of the reaction obtained previously at 453 K and 3 h, we tuned the reaction time from 12 h to 0.08 h (5 min), while keeping the other experimental parameters constant. Every final product of the reaction was structurally and morphologically analyzed. All the details of the experimental parameters of the reactions are summarized in Table S1.

All final products obtained are assigned to the monoclinic KLuW (Figure S14). However, when the time of the reaction was increased furthermore to 12 h, the crystalline phase of the obtained product is assigned to a mixture of monoclinic K_2WO_4 (JCPDS 24-0905) and cubic Lu_2O_3 (JCPDS file 43-1021). The same result was also obtained in the MW assisted solvothermal method when no organic surfactants were included in the reaction solution (reaction MW-10).

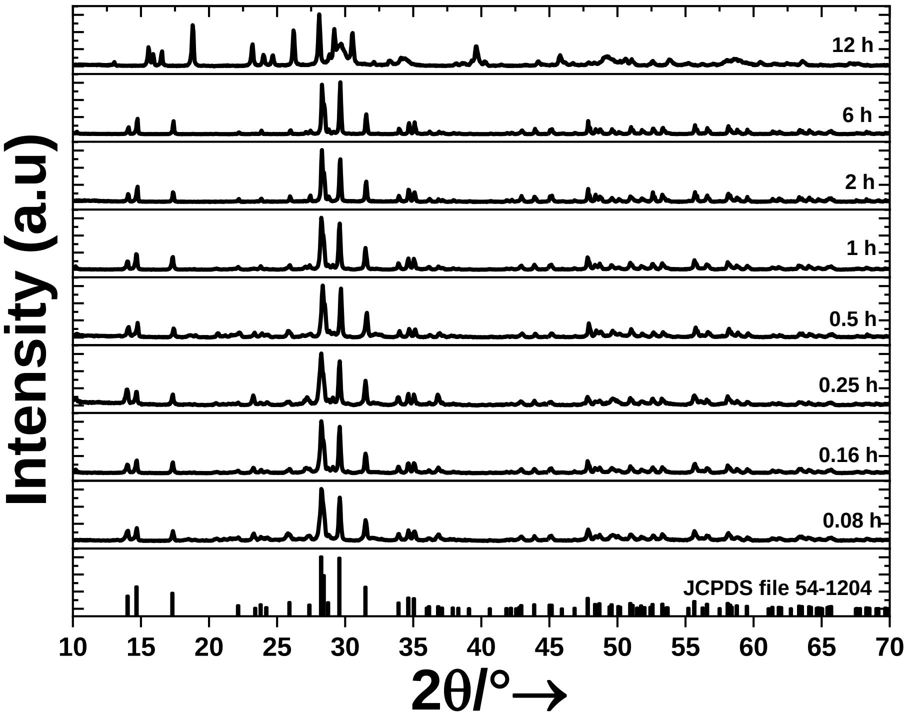

Figure S14. XRPD patterns of the final products obtained by the CA solvothermal at different reaction times. The reference pattern of monoclinic KLuW (JCPDS 54-1204) is included for comparison.

All the morphological features of the final products obtained via the CA solvothermal synthesis tuning the reaction time, are presented in Figure S15 (a)-(h). When KLuW is obtained as a product, in general it exhibits an irregular morphology (see Figure 15b-h) and a tendency to increase its size when the reaction time was different from 3 h (check Table S1 at Supporting Information). Thus, for the reaction performed during 6 h (see Figure S15 (b)), an average size distribution in the range of 1.2 µm was detected. The reactions performed during 2 h (see Figure S15 (c)) and 1 h (see Figure S15 (d)), produced particles with an average size of 142 nm and 171 nm (see Table S2), respectively. Further decrease of the reaction time, resulted in a further increase of the average size of the particles. For example, the reactions at 0.5 h (see Figure S15 (e)) and 0.25 h (see Figure S15 (f)) resulted in 212 nm and 215 nm average size (see Table S2), respectively. An additional decrease of the reaction time to 0.16 h (see Figure S15 (g)), produced particles with an average size of 221 nm (see Table S2). The highest increase of size of the final products, was obtained at the lowest reaction time tested (0.08 h) (see Figure S15 (h)), giving an average size of 416 nm (see Table S2). Thus, the optima conditions for the synthesis of KLuW nanocrystals involve using a reaction temperature of 453 K during 3 h, followed by a calcination at 1023 K during 2 h.

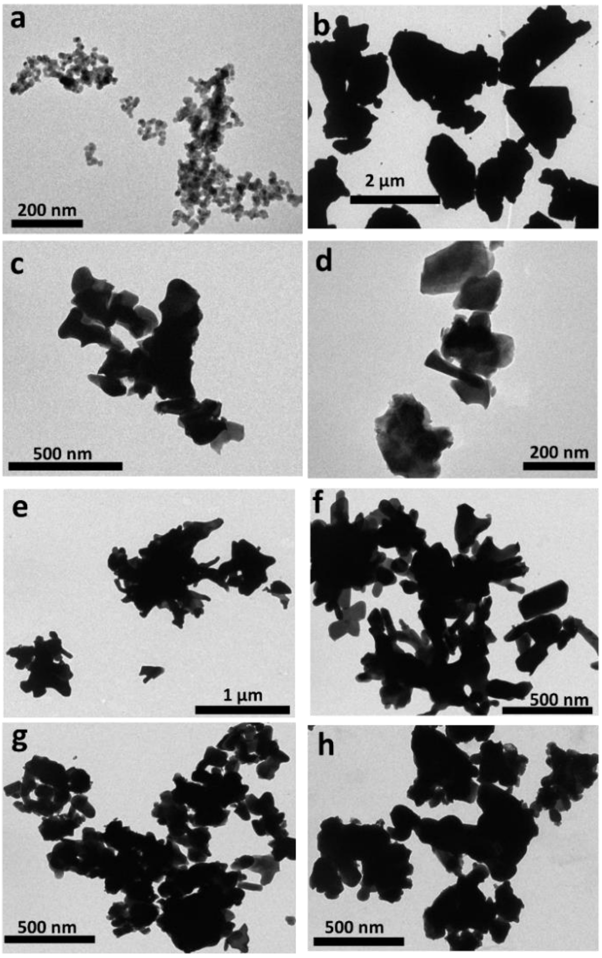

Figure S15. TEM images of the final products obtained by the CA solvothermal at different reaction times: (a) 12 h, (b) 6 h, c) 2 h, (d) 1 h, (e) 0.5 h, (f) 0.25 h, (g) 0.16 h and (f) 0.08 h.

Section S3. Other characterizations of Ho, Tm doped KLu(WO₄)₂ nanocrystals

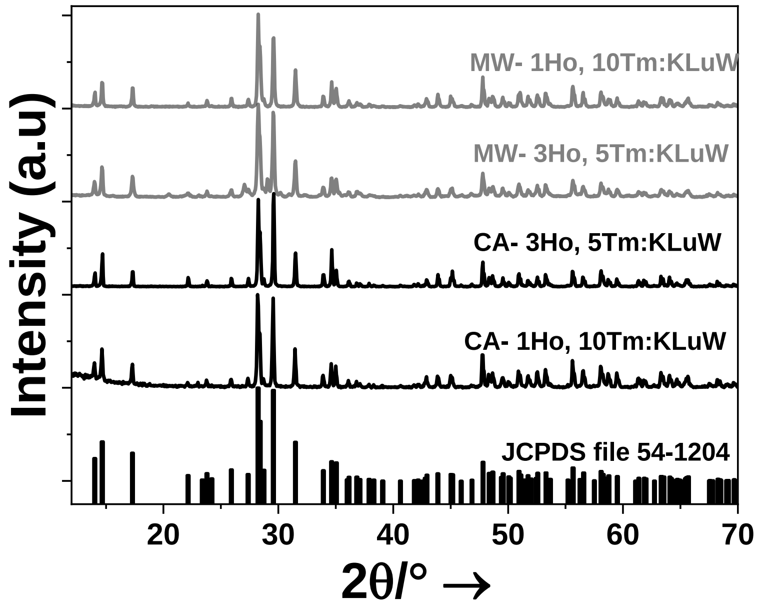

Figure S16. XRPD patterns of 1 mol% Ho, 10 mol% Tm and 3 mol% Ho, 5 mol% Tm: KLuW synthesized via MW and CA solvothermal based methodologies.

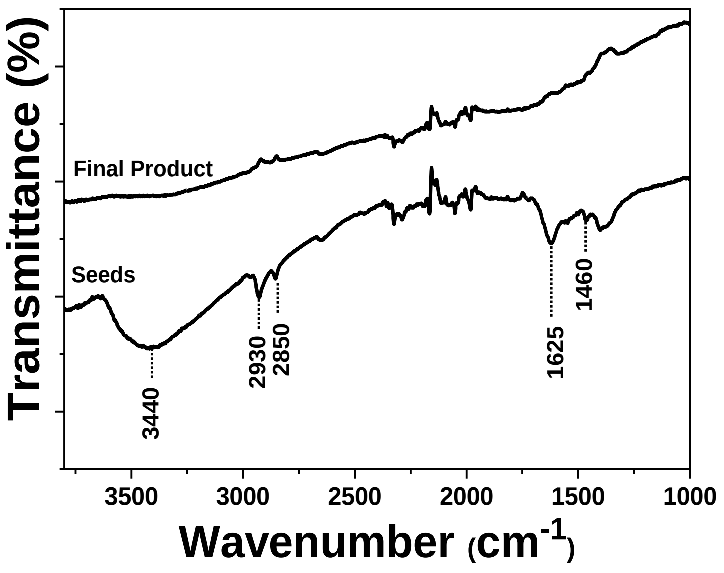

Figure S17. FT-IR spectra of the seeds and KLuW nanocrystals synthesized via the MW solvothermal method.

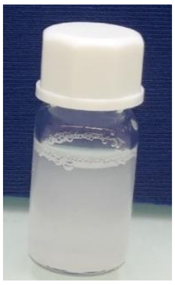

Figure S18. Monoclinic KLuW nanocrystals dispersed in distilled water.

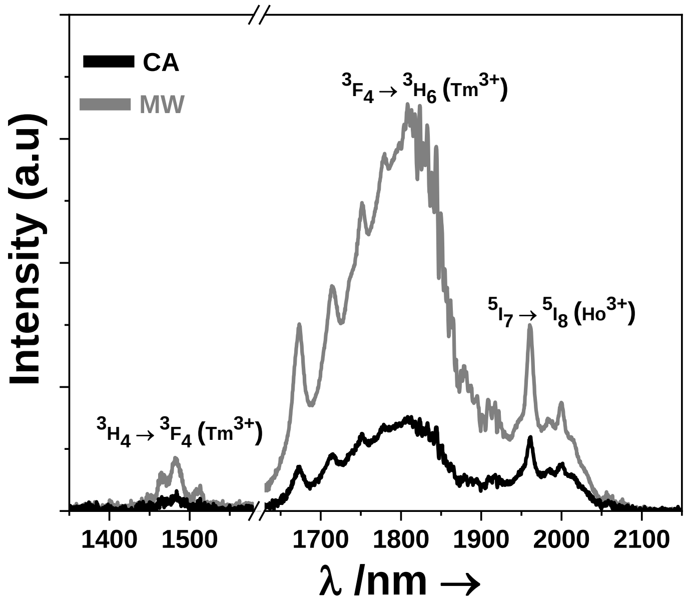

Figure S19. Third biological window emissions recorded at room temperature for 1 mol% Ho, 10 mol% Tm:KLuW materials excited at 808 nm. Please note that part of the graph from 1580 nm to 1630 nm was removed due to the second harmonic of the 808 nm laser source.

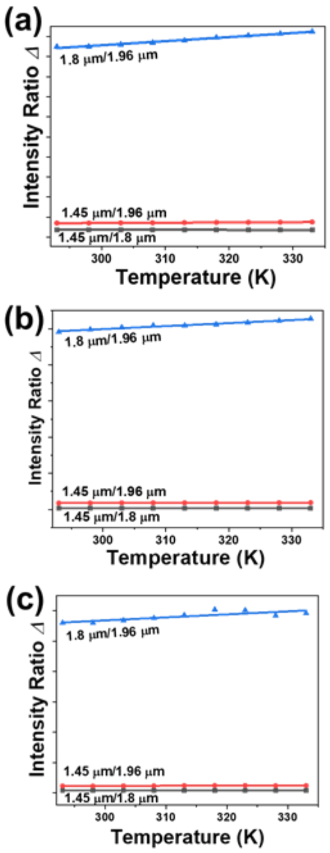

Figure S20. Temperature dependence of the three emission bands in: (a) 3 mol% Ho, 5 mol% Tm:KLuW of MW, (b) 1 mol% Ho, 10 mol% Tm:KLuW of MW, and (c) 1 mol% Ho, 10 mol% Tm:KLuW of CA solvothermal synthesis.

Table S3. Fitting parameters and thermometric performance extracted from the intensity ratio 1.8 μm/1.96 μm for Ho, Tm:KLuW materials synthesized via the solvothermal methodologies

**Sample**	**Synthesis**	**B**	C	ΔE_1-ΔE_2 **(cm^{-1})**	**R^2**	S_{abs} **(K^{-1})**	S_{rel} **(% K^{-1})**	δT **(K)**
1 mol% Ho, 10 mol% Tm	MW	8.5 ± 0.3	159.7 ± 9.9	111	0.98	0.0091	0.18	2.6
1 mol% Ho, 10 mol% Tm	CA	5.1 ± 0.7	172.7 ± 44.8	120	0.99	0.0056	0.20	2.4
3 mol% Ho, 5 mol% Tm	MW	3.7 ± 0.1	198.9 ± 10.8	138	0.98	0.0044	0.23	2.1
3 mol% Ho, 5 mol% Tm	CA	4 ± 0.1	284.9 ± 10.1	198	0.99	0.0051	0.33	1.5

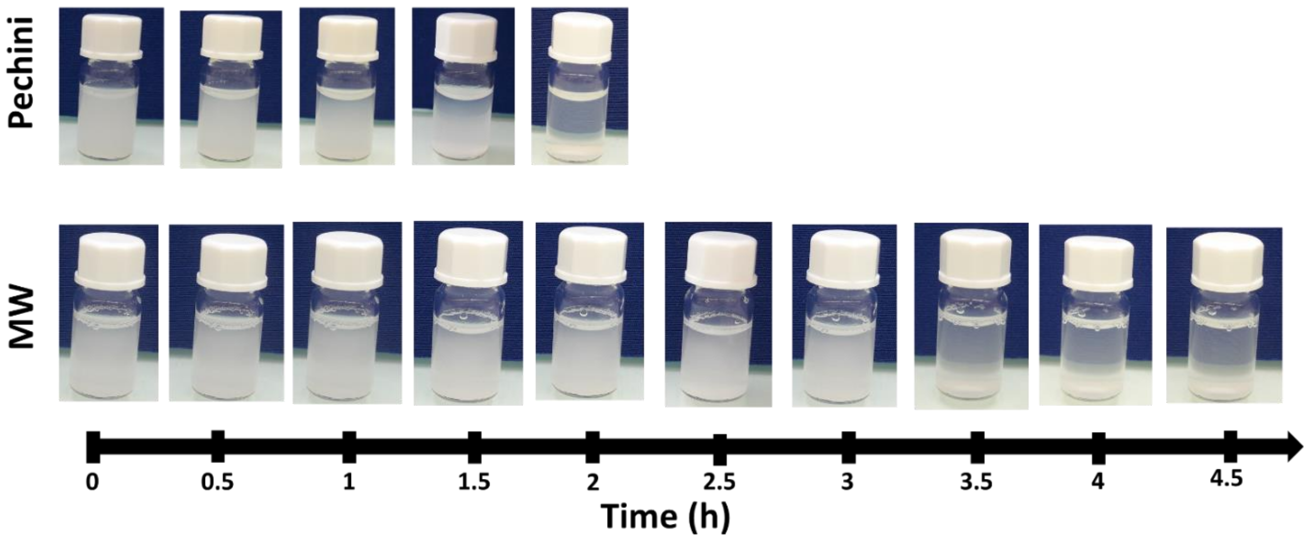

Figure S21. Sedimentation test of Ho, Tm:KLu$(WO_4)_2$ nanocrystals synthesized from the modified sol-gel Pechini and solvothermal (MW as an example) methodologies

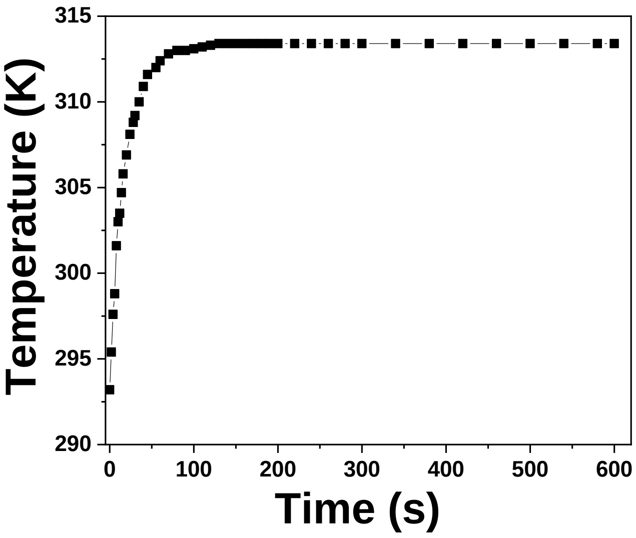

Figure S22. Evolution of the temperature with time for 1 mol% Ho, 10 mol% Tm:KLuW nanocrystals embedded in a 2 mm thick chicken breast piece of meat, recorded with the reference thermocouple.

Section 4. Modelling the temperature dependence of the thermometric parameter Δ

The temperature dependence of the thermometric parameter is based on the model for dual emitting centers and obeys to the Boltzmann law.[1] The methodology is based on the discussion of Cooke *et al*.[2] that extends the classical Mott-Seitz model [3],[4] towards the probability of the radiative and nonradiative processes to take place within a luminescence center. This model describes the total transition probability of an emitting level by the sum of radiative (A_R) and nonradiative (A_{NR}) transition probabilities, which can be correlated to the inverse of the lifetime (τ) of the emitting center as follows:

$$\frac{1}{\tau} = \frac{1}{\tau_R} + \frac{1}{\tau_{NR}} \qquad (1)$$

where τ_R is the radiative lifetime and is assumed to be temperature-independent and equal to τ_0, the lifetime intensity at T= 0 K, and τ_{NR} is the nonradiative lifetime which is expressed by the Arrhenius dependence of the form:

$$\tau_{NR} = \tau_{NR}(0)\exp\left(\frac{-\Delta E}{k_B\, T}\right) \qquad (2)$$

where $\tau_{NR}(0)$ stands for the nonradiative decay time at T= 0 K and ΔE for the activation energy of the thermal quenching process. Based on these equations, Equation 1 can be expressed for τ as follows:

$$\tau = \frac{\tau_0}{1 + \alpha\exp\left(\frac{-\Delta E}{k_B\, T}\right)} \qquad (3)$$

where $\alpha = \frac{A_{NR}}{A_R}$. When more than one quenching process is present, the above expression should be generalized including the deactivation through all the channels:

$$\tau = \frac{\tau_0}{1 + \sum_i \alpha_i \exp\left(\frac{-\Delta E_i}{k_B\, T}\right)} \qquad (4)$$

The integrated luminescence intensity (I) is related to the lifetime by the following equation:[5]

$$\frac{I}{\Gamma_0} = \frac{\tau}{\tau_0} \qquad (5)$$

where Γ_0 is the beam intensity at T= 0 K. Combining Equations 4 and 5, the integrated luminescence intensity can be expressed as:

$$I = \frac{\Gamma_0}{1 + \sum_i \alpha_i \exp\left(\frac{-\Delta E_i}{k_B\, T}\right)} \qquad (6)$$

The thermometric parameter is defined as:

$$\Delta = \frac{I_1}{I_2} \qquad (7)$$

which, taking into account Equation 6 can be expressed as:

$$\Delta = \frac{I_1}{I_2} = \frac{\Gamma_{01}}{1 + \sum_i \alpha_{1i}\exp\left(\frac{-\Delta E_{1i}}{k_B\, T}\right)} \, \frac{1 + \sum_i \alpha_{2i}\exp\left(\frac{-\Delta E_{2i}}{k_B\, T}\right)}{\Gamma_{02}} = \Delta_0 \frac{1 + \sum_i \alpha_{2i}\exp\left(\frac{-\Delta E_{2i}}{k_B\, T}\right)}{1 + \sum_i \alpha_{1i}\exp\left(\frac{-\Delta E_{1i}}{k_B\, T}\right)} \qquad (8)$$

where $\Delta_0 = \frac{\Gamma_{01}}{\Gamma_{02}}$, α_{1i} and α_{2i} stand for the ratios between the nonradiative and radiative probabilities of the i deactivation channel of transitions with intensity I_1 and I_2, and ΔE_{1i}, and ΔE_{2i} are the activation energies for the thermal quenching process of transitions with intensity I_1 and I_2, respectively.

References:

1. Brites, C.D.S.; Millán, A.; Carlos, L.D. Chapter 281-Lanthanides in Luminescent Thermometry. In *Handbook on the Physics and Chemistry of Rare Earths*, Jean-Claude, B., Vitalij K, P., Eds. Elsevier: 2016; Vol. 49, pp. 339-427.
2. Cooke, D.W.; Muenchausen, R.E.; Bennett, B.L.; McClellan, K. J.; Portis, A. M. Temperature-dependent luminescence of cerium-doped ytterbium oxyorthosilicate, *J. Lumin*. **1998**, *79*, 185-190.
3. Mott, N.F. On the absorption of light by crystals. *Proc. R. Soc. A* **1938**, *167*, 384-391.
4. Seitz, F. An interpretation of crystal luminescence. *Trans. Faraday Soc.*, **1939**, *35*, 74-85.
5. Duarte, M.; Martins, E.; Baldochi, S.L.; Vieira, N.D.; Vieira, M.M.F. De-excitation mechanisms of $BaLiF_3:Co^{2+}$ crystals. *Opt. Commun.* **1999**, *159*, 221-224.
